# The mitogenome of *Onchocerca volvulus* from the Brazilian Amazonia focus

**DOI:** 10.1590/0074-02760150350

**Published:** 2016-01

**Authors:** James L Crainey, Túllio RR da Silva, Fernando Encinas, Michel A Marín, Ana Carolina P Vicente, Sérgio LB Luz

**Affiliations:** 1 Fundação Oswaldo Cruz, Instituto Leônidas e Maria Deane, Laboratório de Ecologia de Doenças Transmissíveis na Amazônia, Manaus, AM, Brasil; 2 Fundação Oswaldo Cruz, Instituto Oswaldo Cruz, Laboratório de Genética Molecular de Microorganismos, Rio de Janeiro, Brasil

**Keywords:** *Onchocerca volvulus* mitochondria, onchocerciasis, Brazilian Amazonia focus, mitogenome

## Abstract

We report here the first complete mitochondria genome of *Onchocerca volvulus* from a focus outside of Africa. An *O. volvulus* mitogenome from the Brazilian Amazonia focus was obtained using a combination of high-throughput and Sanger sequencing technologies. Comparisons made between this mitochondrial genome and publicly available mitochondrial sequences identified 46 variant nucleotide positions and suggested that our Brazilian mitogenome is more closely related to Cameroon-origin mitochondria than West African-origin mitochondria. As well as providing insights into the origins of Latin American onchocerciasis, the Brazilian Amazonia focus mitogenome may also have value as an epidemiological resource.

*Onchocerca volvulus* is the sole cause of onchocerciasis in humans, and humans are the parasites’ only known reservoir (Crainey et al. 2016, unpublished observations). The Amazonia onchocerciasis focus is the last of the Latin American foci where *O. volvulus* transmission is still considered to be on-going and as such poses the principal source of risk for new infections (Luz et al. 2014, Crainey et al. 2016, unpublished observations). As mitochondria usually occur in very high copy number within animal cells, they have become a popular target for both parasite and vector molecular diagnostics, taxonomy, and population studies (Post et al. 2009, Conceição et al. 2013, Crainey et al. 2014). The availability of a complete *O. volvulus* mitogenome from South America provides a unique opportunity to characterise single nucleotide variant (SNV) *loci* as well as glean new insights into the parasites’ epidemiological and evolutionary history.

The *O. volvulus* DNA used in this study derived from a skin biopsy taken from a resident of the Brazilian Amazonia focus; parasite DNA was isolated from*O. volvulus* following procedures described previously (Morales-Hojas & Post 2000, Ta Tang et al. 2010). Whole-genome sequencing was performed on an Illumina HiSeq 2500 system (Oswaldo Cruz Foundation, high-throughput sequencing platform) using 2 x 100 bp paired-end reads generated with Nextera Truseq libraries. Reads corresponding to human-host DNA were filtered out by mapping them against a human reference genome (accession GCA_000001405.19). An indexed database of*O. volvulus* genomic sequences (RefSeq; National Center for Biotechnology Information) was constructed to align reads that did not map to the human reference sequences. An *O. volvulus* mitochondria complete genome (accession AF015193) originating from West Africa was used as reference to extract mitochondrial reads corresponding to the Brazilian *O. volvulus*mitogenome (mtOvBz). The assembly of the mtOvBz genome was achieved with the A5-miseq pipeline (arxiv.org/abs/1401.5130). Mapping and short read post-processing were performed using Bowtie2 software (bowtie-bio.sourceforge.net/bowtie2) and Samtools utilities (htslib.org/), respectively. Protein-coding genes, rRNAs, and tRNAs prediction were performed using the MITOS server (mitos.bioinf.uni-leipzig.de/index.py) and Arwen software (mbio-serv2.mbioekol.lu.se/ARWEN/) followed by manual validation comparing homolog regions with the mitochondria reference in Artemis (Rutherford et al. 2000). A mitogenome map was generated using the BRIG software package (brig.sourceforge.net). Polymerase chain reaction and direct Sanger sequencing was used to confirm mtOvBz alleles at 45 of the 46 identified SNV*loci* (Table). Primer3 software (bioinfo.ut.ee/primer3-0.4.0) was used to design 14 primers sets for this purpose.

**TABLE:** Polymerase chain reaction primers used to verify Brazilian Amazonia focus alleles at 45 variable *loci*

Forward primer	Reverse primer	Amplicon size (bp)	Coordinates of verified single nucleotide variations
TGTTTCGTGTGGGAGCTTTT	TGCAACTTCCAACCATCAAA	812	226, 13411, 13639, 13665, 13741
TGGGCTCTGCTGAATCTTTT	AACAAACAACCAAACCAGGAA	582	698, 937, 1052
TGTTTTGTTTGATGTTTTGTTTGA	CCACCTAAACCAGCCCAATA	410	2182
GGGTGGTCCTGGTAGGAGTT	ATCCAAACTAGCAGCCCTCA	733	3066, 3306, 3343
TTGCTGGTTTACAGGGTAT	CTTTTCAACGGATCCCAAT	517	3648, 3837
TTGTTGTAGATTTTGATTTTTCTTTG	AAAACTCCCCCAAATCCATC	751	4481, 4735
TGTGGATTAAGGATGTTATTTTAGAGG	AGTTGAACAACTTAACACGAAAAA	507	6079, 6094, 6101, 6155, 6193
TTTCTTTGTTGTGGAGGGATA	AAAAACAAAAATTCAATACCCAAC	709	6748, 6749, 7002, 7032
TTTTAAGTTTGATTTTGGTTTAGGTTG	ATGTGCCAACAAAATTCACC	401	7586, 7596
GTGAGCTGGTAAGGGGGTTT	AACAACTCCACCGGAACAAC	856	8860, 9488
TTTTGACTTTGGTTTGTATGTTTTTA	ATAAATCCCGCCACTAACCA	311	9643, 9644
TCGGTGTGTTTTGCCTGTAG	CACGCTAAGGCTGCCATTTA	789	10303, 10449, 10624, 10688, 10693, 10731, 10756, 10787, 10885
TTTTCTTGGGGATGGATTTT	AATCCAAACGCCCCTAACAT	837	11246, 11324, 11947
GTTGTCTGCAAATAGGATTTGAT	TGCAAACCCCTACCAATAGC	913	12157, 12697

coordinates are all in reference to the first published*Onchocerca volvulus* mitogenome (AF015193). Nucleotide insertions occur immediately after their coordinates.

A total of 71,936 reads were used in the assembly of a single mitochondria genome contig of 13,769 bp, with ~500x sequencing coverage. The mtOvBz genome is the first from Latin America, the first from outside of Africa, and only the second *O. volvulus* mitochondria genome to be completed. This genome is available from GenBank under accession KT599912. Its total GC content is 26.7%, with base composition of 19.3% A, 54% T, 19.9% G, and 6.8% C. It has 36 genes (12 protein-coding, 22 tRNA and 2 rRNA genes) and a 294 bp noncoding AT-rich region. The mtOvBz is identical in gene content and structure to the West African mitogenome and differs from it at just 37 nucleotide positions (Figure). Interestingly, L-rRNA is the gene with most SNV *loci*; the ND5 gene has the next most, followed by the COX1 and COX3 genes. Comparing the Brazilian and West African origin mitogenomes with a mitochondria draft genome from Cameroon (accession HG738213.2) and other publicly available sequences we have been able to identify a further nine variant*loci*, bringing the total number of known/putative variant*loci* to 46. Considering the available sequence from Cameroon, our analysis showed that this mitogenome is missing ~185 nucleotides corresponding to the tail end of the ND4 gene, immediately before the COX1 gene (nucleotide positions 2,076-2,259 within the AF015193 reference genome).

**Figure:**
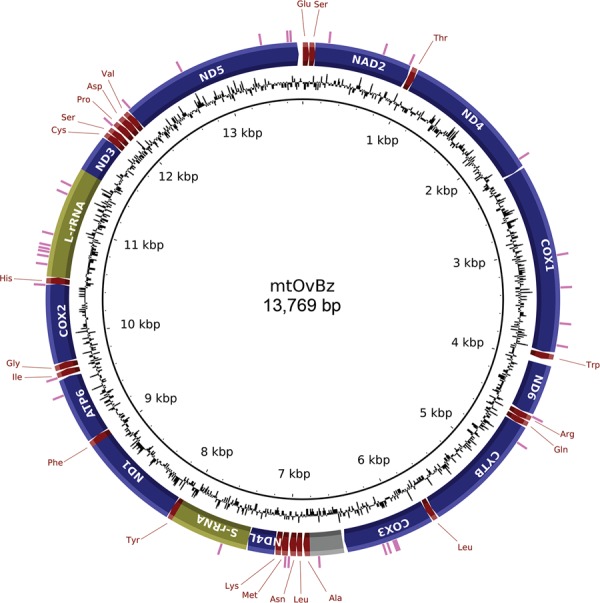
Complete mitogenome of *Onchocerca volvulus* from the Brazilian Amazonia focus (mtOvBz). The 13,769 bp mitogenome structure and content is visualised in a circular map. Protein-coding genes, tRNA, and rRNA genes are depicted in blue, maroon, and olive, respectively. AT-rich region and single nucleotide variations are shown in grey and magenta.

Comparative mitogenomic analysis also revealed that although all *O. volvulus* (West African, Cameroon, and Brazilian Amazonia focus) mitochondria share ~99% of sequence identity, the mtOvBz genome is more closely related to the Cameroon-origin mitogenome than it is to the West-Africa mitogenome. Comparing alleles at the 43 SNV *loci* for which data is available for all three mitogenomes, the mtOvBz genome and the Cameroon-origin mitogenome can be seen to differ at just 20 *loci* whereas the mtOvBz and West African-origin mitogenomes at 36 *loci*. Consistent with the notion that Latin American *O. volvulus* parasites diverged from African parasites after the species radiated in Africa, West African-origin and Cameroon-origin mitogenomes vary at 30 of these 43 nucleotide positions and thus appear more diverged (from one another) than the mtOvBz and Cameroon-origin mitogenomes are (Morales-Hojas et al. 2006, 2007, Morales-Hojas 2009).

The low-levels of genetic variation detected in this study are consistent with previous studies of the parasite’s evolutionary history that have concluded that onchocerciasis is a relatively new form of human parasitism (Keddie et al. 1998, 1999, Morales-Hojas et al. 2006, 2007, Morales-Hojas 2009). The SNVs identified in the present study may represent useful new markers for *O. volvulus*population studies and could therefore provide an important resource to obtain a clearer picture of *O. volvulus* epidemiology to aid with onchocerciasis elimination from the Amazonia focus.
